# *LINC00885* a Novel Oncogenic Long Non-Coding RNA Associated with Early Stage Breast Cancer Progression

**DOI:** 10.3390/ijms21197407

**Published:** 2020-10-08

**Authors:** Martin C. Abba, Romina Canzoneri, Agustina Gurruchaga, Jaeho Lee, Pradeep Tatineni, Hyunsuk Kil, Ezequiel Lacunza, C. Marcelo Aldaz

**Affiliations:** 1 Centro de Investigaciones Inmunológicas Básicas y Aplicadas (CINIBA), Facultad de Ciencias Médicas, Universidad Nacional de La Plata, La Plata CP1900, Argentina; canzonerir@hotmail.com (R.C.); agustinagurruchaga@hotmail.com (A.G.); ezequiellacunza@hotmail.com (E.L.); 2 Department of Epigenetics and Molecular Carcinogenesis, The University of Texas M.D. Anderson Cancer Center, Science Park, Smithville, TX 78957, USA; jhlee@mdanderson.org (J.L.); tatinenip@icloud.com (P.T.); hkil@mdanderson.org (H.K.)

**Keywords:** *LINC00885*, lncRNA, breast cancer, DCIS, proliferation, invasion

## Abstract

Long intergenic non-protein coding RNA 885 (*LINC00885*) was identified as significantly upregulated in breast ductal carcinoma in situ (DCIS). The aim of this study was to characterize the phenotypic effects and signaling pathways modulated by *LINC00885* in non-invasive and invasive breast cancer models. We determined that *LINC00885* induces premalignant phenotypic changes by increasing cell proliferation, motility, migration and altering 3D growth in normal and DCIS breast cell lines. Transcriptomic studies (RNA-seq) identified the main signaling pathways modulated by *LINC00885,* which include bioprocesses related to TP53 signaling pathway and proliferative signatures such as activation of EREG, EGFR and FOXM1 pathways. *LINC00885* silencing in breast cancer lines overexpressing this lncRNA leads to downregulation of proliferation related transcripts such as *EREG*, *CMYC*, *CCND1* and to significant decrease in cell migration and motility. TCGA-BRCA data analyses show an association between high *LINC00885* expression and worse overall survival in patients with primary invasive breast carcinomas (*p =* 0.024), suggesting that the pro-tumorigenic effects of *LINC00885* overexpression persist post-invasion. We conclude that *LINC00885* behaves as a positive regulator of cell growth both in normal and DCIS breast cells possibly operating as a ceRNA and representing a novel oncogenic lncRNA associated with early stage breast cancer progression.

## 1. Introduction

Ductal carcinoma in situ (DCIS) is a late-stage premalignant lesion and non-obligate precursor to most invasive breast carcinomas. It has been estimated that more than one third of DCIS lesions have the potential to progress to invasive ductal carcinoma if left untreated [1]. The reasons why only some DCIS progress to the invasive stage still remain unclear. In a previous study, we performed the first comprehensive molecular profiling of pure high-grade DCIS lesions, thus identifying the main genomic, transcriptomic, methylation and gene pathway changes occurring at this pre-invasive breast cancer stage [2]. RNA-seq profiling allowed us to identify DCIS lesions with the most aggressive phenotypes, based on tumor intrinsic subtypes, proliferative, immune scores and in the activity of specific signaling pathways. Among the transcriptomic signatures of the most aggressive DCIS lesions, we identified the deregulated expression of almost 200 long-non-coding RNAs (lncRNAs), many of which might be associated with breast cancer progression. *LINC00885* was one of the significantly upregulated lincRNAs in aggressive DCIS lesions [2]. LncRNAs are defined as non-coding RNAs exceeding 200 nucleotides in length and without evident protein coding functions [3]. Over ten thousand lncRNAs have been annotated in the human genome, and although they have been increasingly implicated in neoplastic diseases, only a few have been functionally characterized [4]. Based on their location and orientation relative to coding genes lncRNAs can be classified as long-intergenic RNAs (lincRNAs); genic, which include antisense transcripts; divergent lncRNAs run in the opposite strand and direction using the same promoter; promoter associated; and 3′ UTR associated lncRNAs [3]. LncRNAs have been implicated as participating in multiple bioprocesses such as normal development, organogenesis and human disease [4,5]. Among specific molecular functions, lncRNAs have been widely reported to play instrumental roles in gene regulation. They can participate in RNA splicing, editing, localization, stabilization, translation and degradation via a variety of molecular mechanisms [6,7,8,9]. Due to the various processes in which lncRNAs are involved, main challenges are to understand their role in carcinogenesis and evaluate their potential as therapeutic targets. LncRNAs can function either as oncogenes (e.g., *HOTAIR*, *MALAT1*, *H19*) or as tumor suppressors (e.g., *HOTAIRM1*, *NKILA*, *XIST*) by targeting genes that foster or prevent tumor development and progression, respectively [3]. They were shown to act as promoters or inhibitors during breast cancer progression, modulating proliferation, apoptosis, epithelial mesenchymal transition, metastatic dissemination and drug resistance of cancer cells.

Multiple lncRNAs have been reported associated with breast cancer. Thousands of lncRNAs have been shown to be estrogen-regulated in breast cancer lines, most of them acting in cis as eRNAs increasing the expression of estrogen target genes [10,11]. Various lncRNAs have been identified differentially expressed between normal and breast cancer samples or cell lines [12,13,14]. By analyzing breast cancer TCGA RNA-seq datasets subgroups of lncRNAs were identified as associated with prognosis and with specific intrinsic breast cancer subtypes [15,16,17].

Here, we characterized the phenotypic and molecular effects of *LINC00885* expression in non-invasive and invasive breast cancer models while evaluating its role as a potential novel breast cancer driver lncRNA. Overall, we obtained strong evidence to propose this long non-coding RNA as a new oncogene associated with early breast cancer progression.

## 2. Results and Discussion

In a previous study, we identified subgroups of DCIS lesions with differential molecular risk profiles for potential progression to invasive breast cancer [2]. In the subgroup of DCIS with aggressive molecular profiles indistinguishable from invasive carcinomas, we detected the deregulated expression of multiple lncRNAs when compared with normal breast tissue and DCIS with less aggressive molecular profiles. Among the deregulated transcripts we found well-characterized (e.g., *HOTAIR* and *HOTAIRM1*) and novel lncRNAs (e.g., *LINC00885*, *LINC01011*, *LINC01024*) [2]. Therefore, we hypothesize that specific lncRNAs might have a relevant role in promoting the progression of preinvasive lesions to full-blown invasive breast carcinomas. In this study we characterized the molecular and phenotypic effects of *LINC00885* expression in non-invasive and invasive breast cancer models.

### 2.1. LINC00885 Expression and Subcellular Localization in Breast Cancer Lines

**Figure 1:**
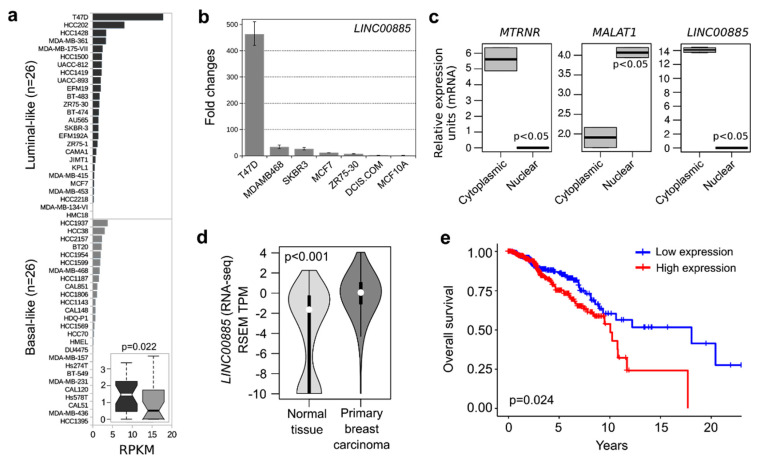
*LINC00885* expression and subcellular localization in breast cell lines. (**a**) Comparative expression profile of *LINC00885* among luminal-like and basal-like breast cancer cell lines based on RNA-seq data obtained from the Broad Institute Cancer Cell Line Encyclopedia (CCLE) available at UCSC Xena resource (https://xenabrowser.net/). RPKM: reads per kilobase of transcript, per million map reads. Insert shows box and whisker plot comparing median and variability of luminal-like cell lines (dark gray) vs. basal-like cell lines (light gray), statistical significance of comparison (*p =* 0.022) indicated. (**b**) Fold changes in *LINC00885* expression levels as determined by RT-qPCR in DCIS and breast cancer cell lines compared to MCF10A normal cell line. All assays were performed in triplicate and normalized to housekeeping gene *GAPDH*. (**c**) Subcellular localization of *LINC00885* in T47D cells. *MALAT1* and *MT-RNR1* were analyzed as nuclear and cytoplasmic markers respectively. Cellular homogenates were separated into nuclear and cytoplasmic fractions and relative *LINC00885* expression was evaluated by RT-qPCR. (**d**) Violin plot showing the *LINC00885* expression distribution (medians as white dots and interquartile as black vertical lines) among normal breast tissue (*n =* 179) and primary invasive carcinomas (*n =* 1092) obtained from TCGA-TARGER-GTEx dataset available at UCSC Xena resource. Mann–Whitney–Wilcoxon test was used to compare the *LINC00885* expression distribution of both groups. (**e**) Kaplan–Meier survival analysis on data of 725 patients obtained from the TCGA-TARGER-GTEx dataset. Breast cancer patients with high *LINC00885* expression (*n =* 360, red line) showed reduced overall survival compared to patients with low expression (*n =* 365, blue line) (log-rank *p =* 0.024).

In silico analysis of *LINC00885* expression among a panel of 52 breast cancer cell lines obtained from the CCLE dataset showed significant upregulation of this transcript in luminal-like breast cancer lines when compared to basal-like cancer lines (Figure 1a, *p =* 0.022). We confirmed some of these observations by RT-qPCR as shown in Figure 1b. The breast cancer line T47D displayed far higher levels of expression compared with other breast cancer lines (e.g., MDA-MB468, SKBR3 and MCF7). DCIS.COM and normal (MCF10A) cell lines displayed low or undetectable *LINC00885* gene expression (Figure 1b). Interestingly, copy number alteration (CNA) analysis of the CCLE dataset showed *LINC00885* gene amplification (at chr3q29 locus) in 11% of human cancer cell lines (100 out of 881) (Supplementary Figure S1a). T47D was the breast cancer cell line most affected by *LINC00885* gene amplification among others (Supplementary Figure S1b). In addition, CNA analysis in the TCGA Pan-Cancer dataset using cBioPortal suggest that *LINC00885* gene amplification is a recurrent alteration in lung squamous cell carcinoma (30% of cases), esophageal (17%), ovarian (16%), cervical (14%) and head and neck cancers (12%) among others (Supplementary Figure S1c).

Tumor-specific up-regulation of some genes can be attributed to aberrant DNA amplification, a phenomenon frequently found in solid tumors. Usually genomic amplification in cancer involves genes that provide some sort of growth advantage or transformation to the cells bearing the amplification. In a previous study, Bing et al., (2017) identified several lncRNAs altered at genomics level in lung cancer. *LINC00885* gene amplification was among the highly altered lncRNAs in 38% of lung squamous cell carcinomas [18]. More recently, Deng et al. (2018) identified 378 candidate driver lncRNAs through integrative analysis of CNA data obtained from TCGA Pan-Cancer dataset [19]. *LINC00885* gene amplification was specifically detected as a potential driver lncRNA that could be associated with the modulation of tumor growth signaling, sustained angiogenesis and metabolic processes in head and neck squamous cell carcinoma (http://biocc.hrbmu.edu.cn/DriverLncRNA/index.jsp).

We characterized the subcellular localization of *LINC00885* transcripts in the T47D breast cancer cell line (Figure 1c). Cellular homogenates were fractionated into cytoplasmic and nuclear fractions. LncRNA *MALAT1* and *MTRNR1* (mitochondrial 12S ribosomal RNA) were used as nuclear and cytoplasmic control markers, respectively. T47D displayed a significant prevalence of *LINC00885* transcripts in the cytoplasmic fraction (*p* > 0.001). Similar to proteins, the function of lncRNAs heavily depends on their subcellular localization. The accumulated lncRNAs in the nucleus may take part in nuclear organization or modulating the epigenetic state of particular genes and participating in transcriptional regulation and alternative splicing. In the cytoplasm, lncRNAs regulate gene expression mainly at post-transcriptional level. On the one hand, lncRNAs can facilitate mRNA decay, stabilize mRNAs, and promote or inhibit the translation of target mRNAs through extended base-pairing [20]. On the other hand, lncRNAs can also function as the microRNAs precursor or compete for microRNA-mediated inhibition, as is the case for the competing endogenous RNAs (ceRNA) [20].

### 2.2. LINC00885 Overexpression Correlates with Poorer Breast Cancer Outcome

*LINC00885* expression was evaluated among normal and breast cancer samples obtained from the TCGA-TARGET-GTEx project (*n =* 1271). As can be observed in Figure 1d primary invasive breast carcinomas showed consistent up-regulation of *LINC00885* when compared with normal samples (*p* < 0.001) (Figure 1d). In order to assess whether *LINC00885* overexpression was associated with cancer progression or patient outcome, *LINC00885* gene expression profiles from 1092 breast cancer patients were grouped into low or high *LINC00885* expression levels. As shown in Figure 1e, Kaplan–Meier analysis revealed that the subgroup of patients with high *LINC00885* expression is associated with a shorter overall survival (median survival = 10 years) compared with those with low expression (median survival = 18 years) (*p =* 0.024). These data suggest that *LINC00885* expression may influence breast cancer progression remaining up-modulated in primary invasive carcinomas. More importantly, evaluation of *LINC00885* expression levels in primary breast cancer samples could be useful as a biomarker of patient prognosis and outcome.

### 2.3. LINC00885 Overexpression Promotes In Vitro Proliferation, Migration, and Invasion of DCIS and Normal Breast Cancer Cells

**Figure 2:**
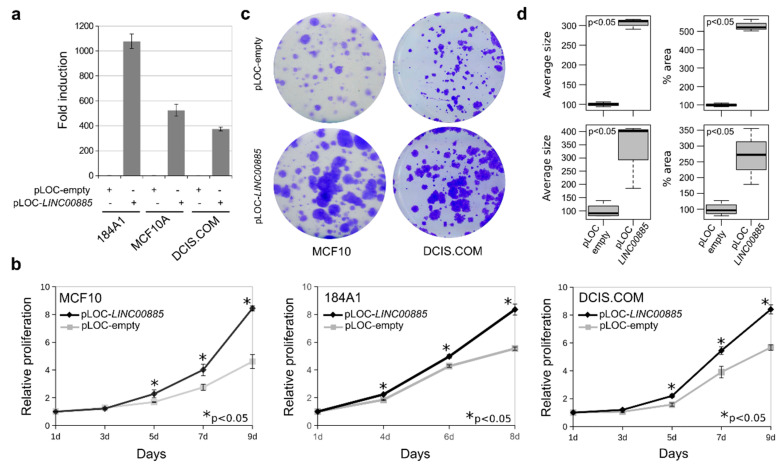
Stable overexpression of *LINC00885* induces increased cell proliferation and colony growth in normal and DCIS breast cell lines. (**a**) Levels of *LINC00885* expression in stably transduced normal breast cell lines (MCF10A and 184A1) and DCIS.COM cell line. Levels of expression were determined by means of RT-qPCR in pLOC-*LINC00885* or pLOC-empty transfected cells (+). All assays were performed in triplicate and normalized to housekeeping gene *GAPDH*. (**b**) Stable overexpression of *LINC00885* increases cell proliferation in normal breast and DCIS cells (* *p* < 0.05). Cells were plated at 1000 cells per well on 96 well plates in triplicate and cell proliferation was determined by means of the colorimetric MTT assay and measuring optical density (OD). (**c**) Cells stably transduced with lentivirus expressing *LINC00885* or vector control were plated at clonal density in 6-well plates. Cells were allowed to grow for 9 days, fixed and stained with crystal violet. (**d**) Box and whisker plots display increased colony size (left) and area occupied by colonies (right) for *LINC00885* stably transduced cells (MCF10 on the top and DCIS.COM on the bottom) compared with vector control. Statistical significance was determined using Mann–Whitney–Wilcoxon test (*p* < 0.05).

To investigate the phenotypic impact of *LINC00885* overexpression on cell growth, migration and invasion, we conducted cell proliferation, colony formation, wound-healing, transwell migration and 3D growth assays on stably transduced breast cells. To this end the full length *LINC00885* (NR_034088.1) cDNA, which is encoded by three exons with locus at human chr3q29, was synthesized and subcloned into a pLOC lentiviral expression vector and virus particles were produced using packaging line Lenti-X 293T. Normal breast cells MCF10A and 184A1 as well as DCIS.COM cells were transduced with a lentiviral expression vector encoding *LINC00885* (pLOC-*LINC00885*) or empty vector control (pLOC-empty) and stable cell lines selected with blasticidin. *LINC00885* overexpression was validated by RT-qPCR in all cell lines (*p* < 0.01, Figure 2a).

We first determined the effects of stable *LINC00885* expression on cell proliferation by means of the MTT assay in MCF10A, 184A1 and DCIS lines. As can be observed in Figure 2b stable *LINC00885* expression behaved as a pro-oncogenic stimulus inducing increased cell proliferation in normal breast cells (MCF10 and 184A1) as well as in the DCIS cell line (*p* < 0.05).

**Figure 3:**
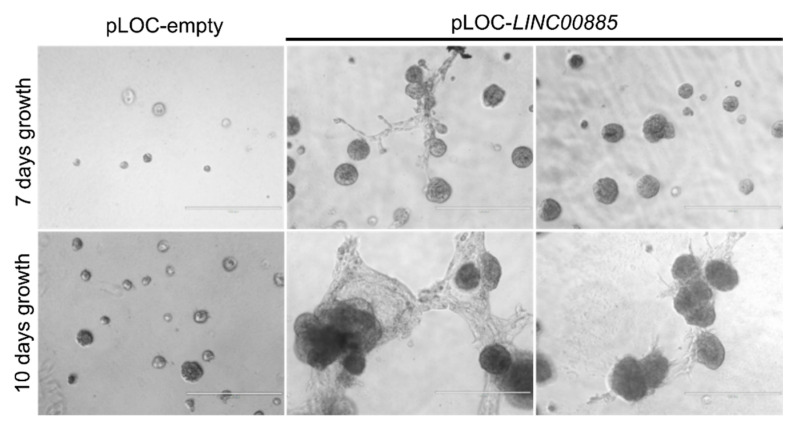
Increase in 3D growth and branching of normal breast cells upon stably expressing *LINC00885*. MCF10A after plating at clonal density increase growth effects were evident when compared with vector control at 7 and 10 days of growth in Matrigel as indicated. Scale bar = 400 µm

The positive effect of *LINC00885* on cell proliferation was further confirmed by means of colony formation assays. MCF10A and DCIS.COM cells stably transduced to overexpress *LINC00885* displayed dramatic increase in colony growth when seeded at clonal density (Figure 2c). Both cell lines showed increased colony average size (*p* < 0.05) and percentage area covered by colonies (*p* < 0.05) indicating increased cell growth (cell proliferation) as consequence of *LINC00885* overexpression (Figure 2c,d). Furthermore, MCF10A were also characterized by effects on 3D growth in matrigel. A significant increase in cell growth and branching was observed for *LINC00885* overexpressing cells (Figure 3).

**Figure 4:**
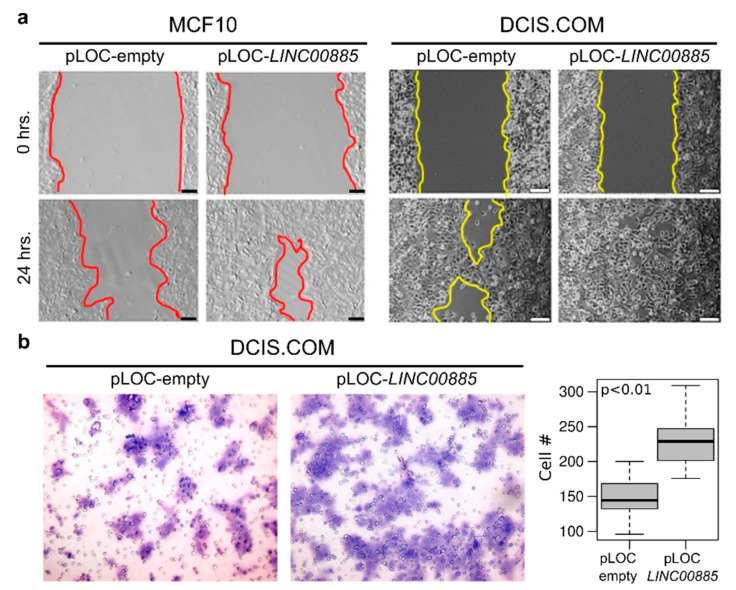
*LINC00885* overexpression increases motility and invasion in normal breast and DCIS cells. (**a**) MCF10A and DCIS.COM stably transduced cells with either vector control or lentivirus expressing *LINC00885* were compared using the in vitro wound-healing assay. As can be observed in representative images, 24 h. after the original scratch the area covered by migrating cells from the edges was compared in both conditions and cell lines. Scale bar = 20 µm. (**b**) Transwell migration assay of DCIS.COM cells stably transduced with *LINC00885*. On left comparative pictures of cells that migrated through the membrane, on the right box and whisker plot of numbers of cells per membrane (*p* < 0.01). Statistical significance was determined using a Mann–Whitney–Wilcoxon test.

To assess the effects of *LINC00885* overexpression on cell motility, we conducted standard wound-healing assays. Normal and DCIS cells stably transduced for *LINC00885* overexpression display increased motility in the in vitro wound-healing assay compared with control cells (*p* < 0.01) (Figure 4a). In additional studies we performed a migration assay with DCIS.COM cells stably transduced with *LINC00885* encoding lentivirus. DCIS cells stably overexpressing *LINC00885* display increased migration as determined by using the transwell migration assay (*p* < 0.01) (Figure 4b). 

**Figure 5:**
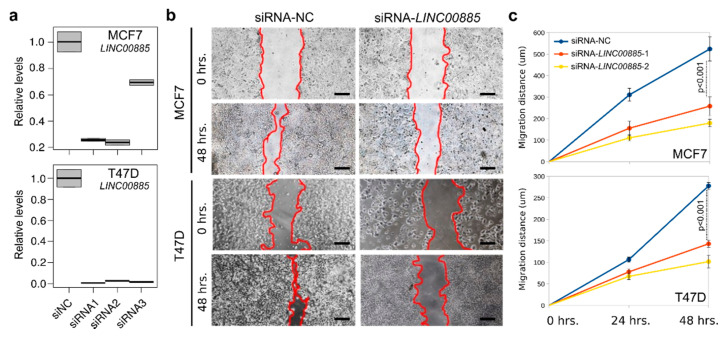
Silencing of *LINC00885* expression significantly decreases cell motility in breast cancer lines. (**a**) Relative *LINC00885* RNA levels in MCF7 and T47D cells that were transiently silenced for *LINC00885* by means of siRNA (siRNA1-3) and compared with scrambled non-specific siRNA control (siNC). (**b**) A significant decrease in cell motility upon *LINC00885* silencing was observed in both breast cancer lines using the wound-healing assay. Forty-eight hours after the original scratch the area covered by migrating cells from the edges was compared. Scale bar = 400 µm (**c**) Graphs showing the comparative effects of negative control siRNA (blue line) and specific *LINC00885* siRNAs (siRNA1 in red and siRNA2 in yellow) on migration distance in wound-healing assays.

We also evaluated the effects of *LINC00885* silencing in breast cancer lines that spontaneously overexpress this lncRNA (T47D and MCF7) in the wound-healing assay. Three independent siRNAs were tested using the TriFECTa RNAi system from IDT. As can be observed in Figure 5a, all three tested siRNAs were extremely effective in silencing expression of *LINC00885* RNA. *LINC00885* depletion in MCF7 and T47D cells significantly decreased the wound closure rate compared to non-target controls (*p* < 0.05, Figure 5b,c) corroborating the gain-of-function studies.

The described results demonstrate that *LINC00885* indeed behaves as a positive regulator of cell growth and migration both in normal and DCIS cells as well as in breast cancer cells.

Given the in vitro results, we proceeded with in vivo phenotyping studies with the cell lines stably transduced. To this end we used intra-mammary fat pad inoculations with cell lines MCF10A and DCIS.COM in SCID mice to compare the in vivo behavior of cells expressing *LINC00885* respect to vector control. As expected MCF10A vector control cells did not show any in vivo growth and MCF10A *LINC00885* also failed to produce any in vivo growth. We performed the same experiment using the DCIS.COM cell line, not detecting any statistically significant differences in in vivo tumor growth comparing *LINC00885* expressing cells vs. vector control (data not-shown). These data suggest that *LINC00885* overexpression may behave as a co-driver of growth in vivo, not being sufficient to exert dramatic differences by itself.

### 2.4. Transcriptome Analysis and RNA-Protein Interaction of LINC00885 Overexpressing Cells

**Figure 6:**
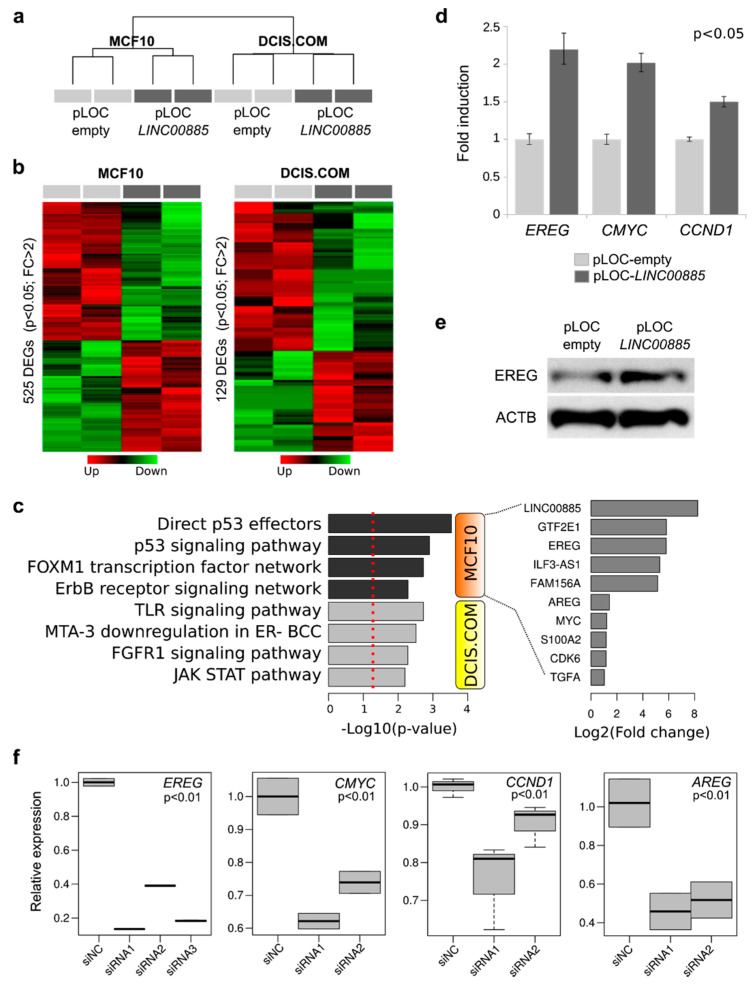
Transcriptomic analysis of *LINC00885* overexpressing cells. (**a**) Hierarchical clustering of MCF10A and DCIS.COM stably transduced cells with either vector control (pLOC-empty) or lentivirus expressing *LINC00885* (pLOC-*LINC00885*) based on RNA-seq profiles. (**b**) Heat map representation of the differentially expressed genes (DEG) obtained by RNA-seq analysis (*p* < 0.05; FC > 2). Red represents upregulated genes and green down-modulated genes. (**c**) Functional enrichment of bioprocesses identified as affected by expression of *LINC00885* in MCF10A and DCIS.COM cells. On the left bioprocesses enriched due *LINC00885* overexpression in both cell lines. The red dotted line represent the basic significance level (*p* < 0.05). At right specific genes identified by RNA-seq analysis and upregulated in MCF10A *LINC00885* expressing cells relative to vector control as Log2 FC. (**d**) RT-qPCR validation of cell proliferation related transcripts (*EREG*, *CMYC* and *CCND1*) in MCF10A *LINC00885* expressing cells compared to vector control (*p* < 0.05). (**e**) Western blot analysis of EREG protein expression in MCF10A cells overexpressing *LINC00885* and vector control. (**f**) RT-qPCR validation of cell proliferation related transcripts in T47D (*EREG* and *CMYC*) and MCF7 cells (*CCND1* and *AREG*) transiently silenced for *LINC00885* and compared with negative control siRNA (*p* < 0.01). All assays were performed in triplicate and normalized to housekeeping gene *RNA18S*. Statistical significance was determined using Mann–Whitney–Wilcoxon test.

In order to better understand the mechanism of action of *LINC00885* we performed RNA-seq studies to analyze the transcriptome of normal breast cells and DCIS cells stably transduced for *LINC00885* overexpression. RNA-seq analysis of MCF10A cells identified 525 differentially expressed genes (DEG) of which 233 were upregulated and 291 were downregulated comparing *LINC00885* expressing cells with the empty-vector cells (FC > 2; *p* < 0.05; Figure 6a). In DCIS.COM cells *LINC00885* overexpression caused the deregulation of 129 genes (52 up and 77 down; FC > 2, *p* < 0.05). Functional enrichment of DEG in MCF10A cells indicated an association with signaling pathways related to the P53 family (TP53, TP63 and TP73), ERBB receptor signaling and FOXM1 signaling. While DCIS.COM deregulated genes showed a significant enrichment of FGF pathway signaling and the downstream JAK/STAT effectors (Figure 6b). *GTF2E1* (*General Transcription Factor IIE subunit 1*), *EREG* (*Epiregulin*), *AREG* (*Amphiregulin*); *MYC* (*MYC proto-oncogene*), *CDK6* (*Cyclin Dependent Kinase 6*) and *TGFA* (*Transforming Growth Factor Alpha*) were among the most representative upregulated genes in MCF10A cells (Figure 6c; Supplementary data 1). We confirmed *EREG*, *CMYC* and *CCND1* upregulation as a consequence of *LINC00885* overexpression by RT-qPCR in normal breast cells using an independent batch of MCF10A stably transduced cells (Figure 6d). Additionally, we also confirmed upregulation of EREG protein by immunoblot in MCF10A cells overexpressing *LINC00885* (Figure 6e). Next, we evaluated the effects of *LINC00885* silencing on the expression of pro-oncogenic genes *EREG*, *CMYC*, *CCND1* and *AREG* in T47D and MCF7 cancer cell lines. As can be observed in Figure 6f significant downregulation in expression of the aforementioned genes was observed as a consequence of *LINC00885* silencing (*p* < 0.01). Interestingly, it has been shown that *EREG* contributes to the formation of early stage breast cancer, and its expression is partially regulated by FGFR1 in MCF10A cells [21]. *EREG* was also proposed as a marker for early ovarian cancer development [22]. EREG acts as a ligand of EGFR or ERBB2 receptors leading to activation of the ERK pathway and MAPK cascade which eventually play key roles in cell proliferation and differentiation [23,24,25].

To gain further insight into the molecular mechanism by which *LINC00885* operates, we conducted RNA-Pull down assays in T47D cells after in vitro transcription of *LINC00885* followed by mass spectrometry (MS) to identify proteins that may potentially bind *LINC00885*. The experiment was performed twice and we determined that *LINC00885* does not appear to bind any specific proteins in T47D cells (data not-shown). This result and the fact that *LINC00885* transcripts are predominantly found in the cytoplasmic compartment are in-line with their potential role as a novel ceRNA as was suggested by gene co-expression network analyses [26]. It is well known that mRNA degradation plays an important role in post-transcriptional regulation of gene expression. miRNAs play key roles in mRNA degradation and ceRNAs are able to bind complementary miRNA response elements blocking the binding of miRNAs on its targets RNAs. Thus, ceRNAs have significant influence in oncogenesis and in progression of tumors by regulating mRNA expression.

In addition to the described *LINC00885* gene amplification in multiple tumor types, and likely in part as a consequence of this phenomenon, this lncRNA was recently described as deregulated in transcriptomic studies of hepatocellular, bladder, malignant pleural mesothelioma and breast cancer [14,26,27,28]. The possibility exists that *LINC00885* may behave as ceRNA which could co-drive the oncogenesis process in various tumor types by regulating mRNA expression through the interaction with microRNAs related to cell proliferation, migration and invasion.

## 3. Materials and Methods

### 3.1. Cell Lines, Cell Culture

All cell lines, with the exception of DCIS.COM (see below), were obtained from the American Type Culture Collection (ATCC, Manassas, VA, USA) and validated by DNA fingerprinting. MCF10A cells (ATCC #CRL-10318) were cultured in Dulbecco’s Modified Eagle Medium F-12 (DMEM/F-12, Sigma-Aldrich, St. Louis, MO, USA) supplemented with 5% horse serum, 20 ng/mL epidermal growth factor (Sigma-Aldrich, St. Louis, MO, USA), 100 µg/mL hydrocortisone (Sigma-Aldrich, St. Louis, MO, USA), 10 µg/mL insulin (Sigma-Aldrich, St. Louis, MO, USA), 100 ng/mL cholera toxin (Sigma-Aldrich, St. Louis, MO, USA) and 100 U/mL penicillin - 100 μg/mL streptomycin (Sigma-Aldrich, St. Louis, MO, USA). 184A1 cells were grown in MCDB170 media as previously described [29]. MCF10 DCIS.COM (hereafter DCIS.COM) cells were a kind gift from Dr. Daniel Medina [30] and were maintained in DMEM/F-12 supplemented with 5% horse serum. The DCIS.COM cell line has been extensively used for functional DCIS studies, and derived from a xenograft originating from MCF10AT cells that were injected into severe combined immune-deficient mice. The MCF10AT series of cell lines was originally transformed by HRAS (p.Gly12Val) [31]. MDA-MB-231, MCF7 and T47D cell lines were cultured in DMEM (Sigma-Aldrich, St. Louis, MO, USA) supplemented with 10% of fetal bovine serum (FBS; Natocor, Córdoba, Argentina) and 100 U/mL penicillin—100 μg/mL streptomycin (Sigma-Aldrich, St. Louis, MO, USA). Cell lines were maintained to 37 °C with 5% CO2.

### 3.2. LncRNA Subcellular Localization Methods

To determine the subcellular localization of endogenous *LINC00885* transcripts in breast cancer lines we used the PARIS Kit (Thermo Fisher Scientific, Waltham, MA, USA) to isolate nuclear and cytoplasmic RNA fractions from cultured cells. Briefly, T47D and MCF7 cells were cultured in 10 mm plates as described above. Cells were trypsinized, washed and resuspended in the required amount of lysis buffer. The nuclear and cytoplasmic fractions were separated by centrifugation at 400 g for 1 min at 4 °C. RNA was extracted from the two fractions according to the kit instructions. The expression of *MALAT1* and *MT-RNR1* were determined by RT-qPCR in each fraction as nuclear and cytoplasmic markers respectively.

### 3.3. Stable LINC00885 Expressing Cells

The full-length sequence of *LINC00885* (1828 bp spanning three exons, NCBI Reference Sequence: NR_034088.1) was synthesized (Genscript, Piscataway, NJ, USA), sequenced verified and subsequently cloned into the pLOC lentiviral expression vector. Virus particles were produced using packaging line Lenti-X 293T (Takara Bio, Mountain View, CA, USA). Normal breast epithelial cell lines MCF10A, 184A1 and DCIS cell line DCIS.COM were stably transduced and selected with 10µg/mL blasticidin. *LINC00885* overexpression was confirmed in all cell lines by RT-qPCR.

### 3.4. RNA Interference (siRNA) Assay

*LINC00885* expression was silenced in T47D and MCF7 cells using the TriFECTa Kit DsiRNA Duplex (Integrated DNA Technologies, Coralville, IA, USA). Three siRNA duplexes targeting *LINC00885* transcript were used: siRNA-1 (5′-UAAGAAAUCCCCAUGAUUCCACUGGGG-3′), siRNA-2 (5′-UCCUCAGGUGUUGGUGCUUUAUCUUGG-3′) and siRNA-3 (5′-GAAUCAGAUUAGAUCCAUUCUGCUCAG-3′). A siRNA without target in human transcriptome was included (5′-AUACGCGUAUUAUACGCGAUUAACGAC-3′) as non-target control. Transfection of siRNA duplexes (150 pmol/well) were performed with Lipofectamine 3000 in 6 well plates at 70% confluence cells and incubated overnight. Cell culture medium was replaced by a siRNA free culture medium and cells were incubated 48 h.

### 3.5. RNA Isolation and Real Time Quantitative PCR (RT-qPCR)

Total RNA was isolated from cell lines using TRI Reagent solution (Thermo Fisher Scientific, Waltham, MA, USA) according to manufacturer’s instructions. RNA was reverse transcribed into cDNA using the SuperScript reverse transcriptase kit (Thermo Fisher Scientific, Waltham, MA, USA). Primers were designed for *LINC00885*, *MALAT1*, *MT-RNR1*, *EREG*, *AREG*, *CMYC*, *CCND1* and *RNA18S* (Supplementary data 2). PCR conditions were as follows: an initial denaturation step of 95 °C for 3 min and 40 cycles of 95 °C 40 s, 55–60 °C 30 s and 72 °C 30 s. Data was captured and analyzed using the AriaMx Real Time PCR Software (Agilent Technologies, Santa Clara, CA, USA). Real-time PCR assays were performed using PerfeCTa SYBR Green SuperMix (Quanta BioSciences Inc., Beverly Hills, CA, USA). Experiments were done in triplicate and normalized to *GAPDH* or *RNA18S* expression using the comparative threshold cycle (2^−ΔΔCT^) method. Values were considered as differentially regulated between groups at the *p* < 0.05 statistical significance level.

### 3.6. Cell Proliferation, Clonal Growth, Cell Motility and Migration Assays

MCF10A, 184A1 and DCIS.COM stably transduced to overexpress *LINC00885* or an empty vector control were plated (1000 cells per well) on 96 well plates in triplicate and cell proliferation was determined by means of the colorimetric MTT assay kit (Cell Proliferation Kit, Roche, Basel Switzerland) and measuring optical density (OD).

For clonal growth assays, MCF10A and DCIS.COM stably transduced to overexpress *LINC00885* or vector control were plated at clonal density (500 cells/dish) in individual wells of 6-well plates and maintained in adequate media as described above. After 9 days of growth, cells were fixed and colonies stained with crystal violet. Digital images of individual wells were obtained and used to determine the number and area of growing colonies using ImageJ software.

To assess cell motility, we conducted a standard wound-healing assay. Briefly, 1 × 10^6^ cells were seeded in each well. After cells adhered, the FBS concentration in the medium was reduced to 2% to decrease cell proliferation. Two scratch wounds were made in confluent cell cultures. Images of the same fields were collected at 0, 24 and 48 h. To quantify the cell migration rate, the width of the wound was determined at ten separate sites for each time point. The assay was performed by triplicate, mean and standard deviation were calculated for each determination. Transwell migration assays were performed using standard Boyden chambers containing 12 μm pore divider membranes, 5% FBS was used in the lower chamber as chemoattractant.

### 3.7. Tumorigenicity Assay

MCF10A and DCIS.COM stably transduced cells with *LINC00885* or empty vector as control were inoculated bilaterally intra-mammary fat pad (4th and 9th inguinal mg. fat pads) into female SCID mice at two cell concentrations (2 × 10^6^ and 5 × 10^6^ cells). Tumor growth was monitored, and after an observation period of 25 days, mice were euthanized and, when present, tumors collected.

### 3.8. RNA-Seq Data Analysis

MCF10A and DCIS.COM stably transduced cells were used for RNA isolation from subconfluent plates using the RNeasy kit (Qiagen, Germantown, MD, USA). RNA concentration and integrity were measured on an Agilent 2100 Bioanalyzer (Agilent Technologies, Santa Clara, CA, USA). Only RNA samples with RNA integrity values (RIN) over 8.0 were considered for subsequent analysis. RNA-seq library construction was performed using the ScriptSeq v2 RNA-seq Library Preparation Kit (Epicentre, Madison, WI, USA) according to the manufacturer’s protocol. We performed 76 nt paired-end sequencing using an Illumina HiSeq2000 platform and ~20 million reads per sample were obtained. The short-sequenced reads were mapped to the human reference genome (hg19) by the splice junction aligner Rsubread package. We employed several R/Bioconductor packages to accurately calculate the gene expression abundance at the whole-genome level using the aligned records (BAM files) and to identify differentially expressed genes between cells stably transduced with *LINC00885* and empty vector. Briefly, the number of reads mapped to each gene based on the UCSC.hg19.KnownGene database were counted, reported and annotated using the GenomicFeatures, Rsamtools and org.Hs.eg.db packages. To identify differentially expressed genes (log2 fold change [log2 FC] > ±1, False Discovery Rate [FDR] < 0.05) between the empty vector and *LINC00885* overexpressing counterparts, we utilized the edgeR Bioconductor package based on the normalized log2 based count per million values. For functional enrichment analyses, we used ClueGo Cytoscape’s plug-in (http://www.cytoscape.org/) and the InnateDB resource (http://www.innatedb.com/) based on the list of dysregulated transcripts. Data integration and visualization of differentially expressed transcripts were performed with R/Bioconductor and the MultiExperiment Viewer software (MeV v4.9).

### 3.9. RNA-Pulldown and Mass Spectrometry Analysis

RNA-pulldown assays after in vitro transcription of *LINC00885* followed by mass spectrometry (MS) were performed in T47D cells using the Pierce Magnetic RNA-Protein Pull-Down Kit (Thermo Fisher Scientific, Waltham, MA, USA). Briefly, pU19-*LINC00885* was used to obtain the DNA template for in vitro RNA synthesis using T7 RNA polymerase. This RNA was labeled using T4 RNA ligase for attaching a single desthiobiotinylated cytidine bisphosphate to the 3′ end of the RNA strand. Pull-down was performed using streptavidin beads and MS analyses were performed at the MDACC core facility. These studies were repeated twice including validated positive and negative (Bead-T47G) controls for RNA labeling and pull-down.

### 3.10. Western Blot Analysis

Proteins were isolated from cell lines using TRI Reagent TM solution (Thermo Fisher Scientific, Waltham, MA, USA) according to manufacturer’s protocol. Protein samples were subjected to 12% SDS/PAGE and Western blot analysis (. The primary antibody used was rabbit anti-EREG (1:1000, Origene, Rockville, MD, USA). Secondary antibodies: HRP-conjugated anti-rabbit secondary antibody (1:2000, Dako, Glostrup, Denmark) and HRP-conjugated anti-mouse secondary antibody (1:1000, Dako, Glostrup, Denmark). For antigen detection, we used the enhanced chemiluminescence (ECL) EasySee Western Blot Kit.

### 3.11. In Silico Analysis of LINC00885 in Breast Cancer

*LINC00885* RNA-seq expression profiles among 26 luminal-like and 26 basal-like breast cancer cell lines were obtained from the Broad Institute Cancer Cell Line Encyclopedia (CCLE) available at UCSC Xena resource (https://xenabrowser.net/). To evaluate *LINC00885* expression in normal tissue and primary invasive breast cancer the TCGA-TARGET-GTEx RNA-seq dataset was downloaded from the UCSC Xena resource. ENSG00000224652.1 transcript was compared among GTEx normal breast tissue samples (*n =* 179) and TCGA primary invasive breast carcinomas (*n =* 1092) using the R Wilcoxon–Mann–Whitney test. For overall survival analysis, primary invasive carcinomas were grouped into high or low *LINC00885* expression subgroups based on the upper and lower terciles of the ENSG00000224652.1 transcript expression (RSEM TPM) profile. Statistical analysis was performed using the computing environment R and survival and survminer packages. Copy number alteration (CNA) of *LINC00885* gene was performed in CCLE and TCGA Pan-Cancer datasets using cBioPortal resource (https://www.cbioportal.org/).

## 4. Conclusions

In conclusion, the described results indicate that *LINC00885* overexpression induces premalignant phenotypic changes in normal breast epithelial and DCIS cells by increasing cell proliferation, motility, and migration, and altering 3D growth. However, no significant phenotypic effects were observed in vivo. RNA-seq studies were performed to profile the transcriptome of *LINC00885* overexpressing cells. In agreement with the in vitro observations we identified overexpression of transcripts and pathways associated with cell proliferation in gain and loss-of-function studies. In addition, we determined that *LINC00885* RNA molecules are predominantly found in the cytoplasmic compartment and based on RNA pull-down studies, they do not appear to preferentially bind any protein, suggesting a potential role as a ceRNA lncRNA. Finally, *LINC00885* expression may influence early breast cancer progression affecting patient outcome. Further mechanistic characterization of *LINC00885* in other cancer cell lines and in vivo models may provide insights into how this lncRNA could contribute to breast cancer development and progression. 

## Supplementary Materials

Supplementary materials can be found at https://www.mdpi.com/1422-0067/21/19/7407/s1.

## Abbreviations

DCIS	Ductal carcinoma in situ
lincRNA	Long-intergenic RNA
CNA	Copy number alteration
DEG	Differentially expressed genes
