# Supplementary material for: LINC00885 a Novel Oncogenic Long Non-Coding RNA Associated with Early Stage Breast Cancer Progression
Source: Int J Mol Sci. 2020 Oct 8;21(19):7407. doi: 10.3390/ijms21197407 (PMC7582527; doi:10.3390/ijms21197407)
Supplement: Supplementary file 1 [file ijms-21-07407-s001.zip › Supplementary Data 2.pdf]

Supplementary Data 2. List of primers employed in RT-qPCR analysis.

Gene	Forward 5' > 3'	Reverse 5' > 3'	Amplicon
LINC00885	CCAGCAGGGCCTAGTAACAC	CCTTGCTCTTGGTGAGTGGT	172
MTRNR1	ACACATGCAAGCATCCC	GGCTGGCACGAAATTGA	205
MALAT1	CACCGAAGGCTTAAAGTAGGAC	GCTGACACTTCTCTTGACCTTAG	93
EREG	CCCAGGAGAGTCCAGTGATA	AAGGTTGGTGGACGGTTAAA	221
AREG	GCTGCCTTTATGTCTGCTGTGA	TGGAAAGAGGACCGACTCATC	226
CMYC	CACCAGCGACTCTGA	GATCCAGACTCTGACCTTTTG	102
CCND1	GGATGGAGTTGTCGGTGTAGATG	AGGAACAGAAGTGCGAGGAGG	192
GAPDH	ACAACTTTGGTATCGTGGAAGG	GCCATCACGCCACAGTTTC	294
RNA18S	GTAACCCGTTGAACCCCATT	CCATCCAATCGGTAGTAGCG	151
