# Supplementary figures and images for: LINC00885 a Novel Oncogenic Long Non-Coding RNA Associated with Early Stage Breast Cancer Progression
Source: Int J Mol Sci. 2020 Oct 8;21(19):7407. doi: 10.3390/ijms21197407 (PMC7582527; doi:10.3390/ijms21197407)

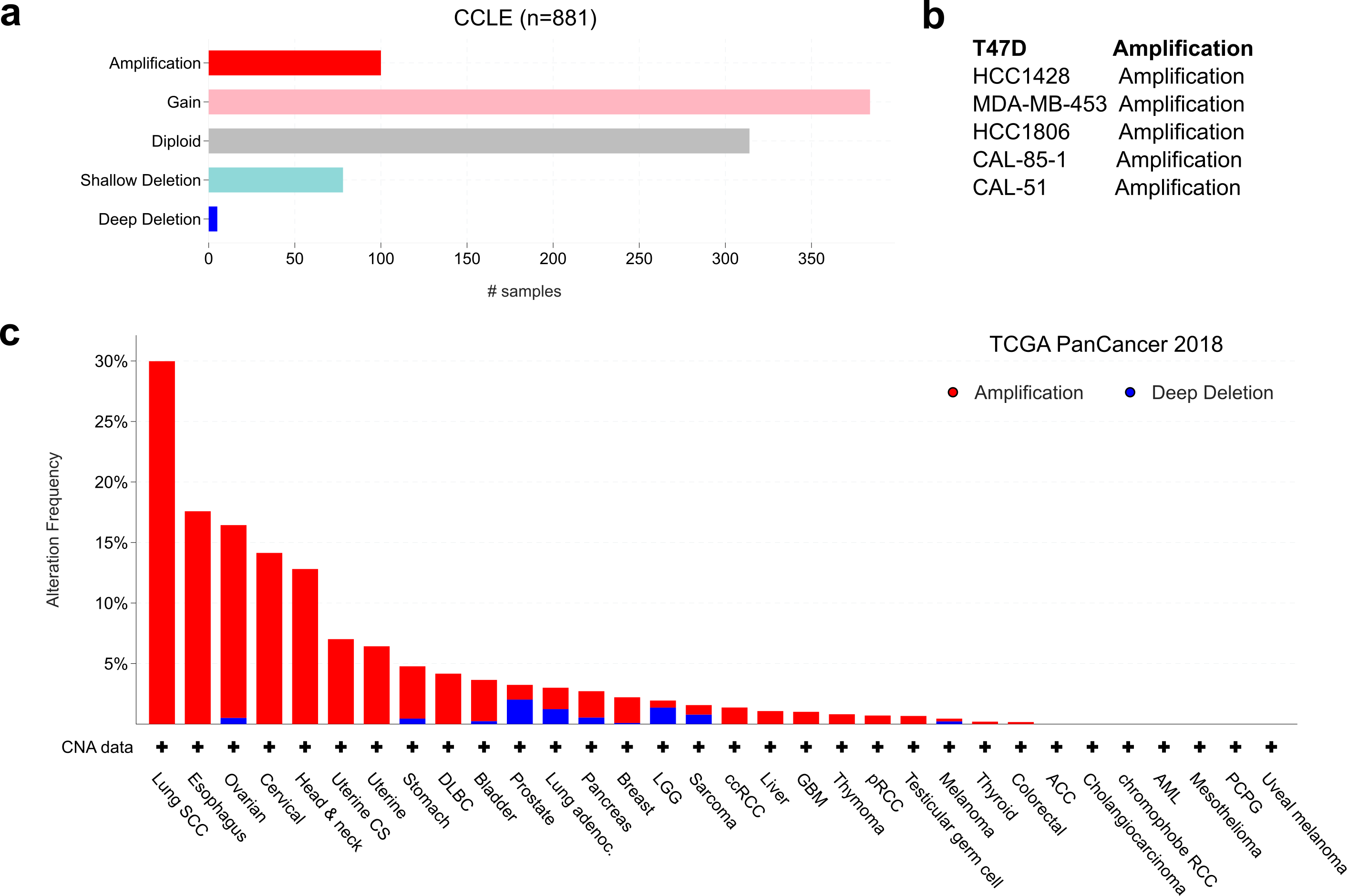

Supplement: Supplementary file 1 [file ijms-21-07407-s001.zip › Figure S1.png]
